# The Coexistence of Cervical Intraepithelial Neoplasia (CIN3) and Adenocarcinoma In Situ (AIS) in LEEP Excisions Performed for CIN3

**DOI:** 10.3390/cancers16050847

**Published:** 2024-02-20

**Authors:** Maria Teresa Bruno, Gaetano Valenti, Nazario Cassaro, Ilenia Palermo, Giosuè Giordano Incognito, Antonino Giovanni Cavallaro, Francesco Sgalambro, Marco Marzio Panella, Liliana Mereu

**Affiliations:** 1 Gynecology and Obstetrics Unit, Department of General Surgery and Medical-Surgical Specialty, Rodolico University Hospital, University of Catania, 95123 Catania, Italy; giordanoincognito@gmail.com (G.G.I.); ninocavallaro@tin.it (A.G.C.); f.sgalambro@policlinico.unict.it (F.S.); mpanella@unict.it (M.M.P.); liliana.mereu@unict.it (L.M.); 2 Multidisciplinary Research Center in Papillomavirus Pathology, Chirmed, University of Catania, 95100 Catania, Italy; valentigaetano@gmail.com (G.V.); cassanaza@hotmail.it (N.C.); 3 Humanitas Medical Care, Gynaecologic Oncology Unit, 95125 Catania, Italy; 4 Virology Unit, Rodolico Polyclinic, 95123 Catania, Italy; palermoilenia77@gmail.com

**Keywords:** coexistent CIN3-AIS, trachelectomy, fertility sparing, specific genotyping

## Abstract

**Simple Summary:**

The aim of this study was to evaluate the incidence of high-grade glandular pathology in the histological cones of LEEP for CIN3 and to verify the different biological nature of the single CIN3 lesion from CIN3 coexisting with AIS. A sample of 414 women underwent LEEP for CIN3. From the histological examination of the cone, we selected 370 women with a single CIN3, 39 women with CIN3 coexisting with AIS, and 5 women with CIN3 coexisting with AC. Specific HR HPV genotypes and treatment outcome in the study groups were investigated. Women with CIN3 coexisting with AIS showed only four specific HR HPV genotypes (16, 18, 45, 33), the prevalence of genotype 18, the presence of skip lesions (9%), and occult adenocarcinoma (one case of 1A1 adenocarcinoma). Furthermore, CIN3 lesions coexisting with AIS have a higher rate of viral persistence and recurrence than single CIN3 lesions. These characteristics make CIN3 coexisting with AIS closer to the glandular pathology than to the squamous one, making hysterectomy necessary and cervical conization insufficient, which is instead considered the optimal treatment method for a single CIN3.

**Abstract:**

The purpose of this study was to evaluate the incidence of AIS and AC in the histological cone of women treated for CIN3. Furthermore, through the study of the specific HR HPV genotypes, we obtained more information on the possible different nature between the single CIN3 lesion and the CIN3 coexisting with the glandular lesion. Methods. A sample of 414 women underwent LEEP for CIN3. The study sample consisted of 370 women with a CIN3 lesion alone and 44 women with a CIN3 lesion coexisting with AIS or adenocarcinoma. We studied the individual HR HPV genotypes and their frequency in the two groups under study. Furthermore, the therapeutic results and follow-ups for the population were studied on the entire study sample. Results. In patients with a single CIN3 lesion, 11 high-risk genotypes were detected; in patients with CIN3 associated with AIS or AC, only 4 different genotypes were detected (16, 18, 45, 33). Overall, the frequency of HPV 18 was significantly higher in CIN3 coexisting with AIS compared to solitary CIN3 lesions, χ2 = 27.73 (*p* < 0.001), while the frequency of other high-risk genotypes was significantly higher in patients with a single CIN3 than in patients with CIN3 coexisting with AIS. In our study population, mixed lesions (CIN3 coexisting with AIS), unlike their squamous counterparts (single CIN3 lesions), were characterized by skip lesions, which demonstrate more aggressive behavior and a higher rate of viral persistence and recurrence. Conclusion. A relatively high rate (10.7%) of AIS-AC was found in women treated for CIN3. Our study confirms the multifocal biological nature of the CIN3 lesion coexisting with AIS compared to the single CIN3 lesion. All this justifies the different treatments to which CIN3 lesions coexisting with AIS are addressed; in fact, the latter are treated with hysterectomy, while CIN3 is treated with conization alone.

## 1. Introduction

In 2018, cervical cancer ranked as the fourth most common cause of cancer incidence and mortality in women worldwide [1]. Squamous carcinoma of the cervix (SCC) represents 80% of cervical carcinoma cases, while invasive adenocarcinoma (AC) represents 20% of cases [2].

The prevalence of HPV in glandular lesions is just as high as in cervical squamous cell carcinoma [3,4].

Our knowledge of the papillomavirus acquired over the years and the diagnostic techniques used, up to the screening and vaccine against HPV, give us hope for defeating cervical carcinoma by 2030 [5,6,7]. In fact, the incidence of SCC is decreasing, at least in the most developed countries; unfortunately, there has not been a similar decrease for AC [8].

Adenocarcinoma in situ (AIS) is the only known precursor to cervical adenocarcinoma; therefore, early diagnosis and management of AIS are critical to prevent invasive adenocarcinoma [9]. While the natural history of squamous pathology is well known, this is not the case for glandular pathology; in fact, AIS represents 2% of all cervical intraepithelial lesions, while AC represents from 6% to 18% of all invasive lesions of the cervix [10]. This indicates that in the natural history of glandular lesions the preinvasive phase is very short [9] or that there is a significant difficulty in diagnosis.

High-risk genotypes were detected in 66–90% of patients with AIS and 99.7% of CIN cases [11].

In 2018, the International Endocervical Criteria and Classification (IECC) divided cervical glandular pathologies into HPV-positive lesions and HPV-negative lesions. Compared to the WHO classification, they suggested a classification based on pathogenesis that is more informative from a clinical point of view [12]. In total, 10–20% of glandular neoplastic pathologies are HPV-negative and have a worse prognosis than HPV-related lesions [9,13].

Both CIN3- and HPV-related AIS arise at the squamocolumnar junction, while non-HPV-related AIS can be located in the upper cervical canal, and both types can be multifocal [14].

The low incidence of glandular neoplasms in the population as well as their endocervical localization and multifocality are the reasons why cytologists and colposcopists rarely gain sufficient experience, making diagnosis difficult. This contributes to the fact that only 50% of AIS cases are correctly diagnosed before conization [15].

Glandular neoplasms often coexist with invasive SCC or CIN3, constituting a “mixed disease”, in particular approximately 10% of all women diagnosed with CIN3 have coexisting glandular lesions and approximately 40% of AIS and AC are diagnosed incidentally following of biopsy or cone performed for CIN3 [16].

The aim of this study was to analyze women treated with LEEP for CIN3, evaluating the rate of associated glandular pathology (AIS or AC), and to study the frequency of specific HR HPV genotypes in patients with a single CIN3 lesion and in patients with a mixed lesion (CIN3 coexisting with AIS or AC) in order to obtain more information on the possible different nature of the mixed lesion (CIN3 coexisting with AIS) from a single squamous lesion (CIN3).

## 2. Materials and Methods

This is a retrospective and multicenter study. Data from 491 patients who underwent LEEP for CIN between 2015 and 2021 were collected in three second-level centers for the diagnosis and treatment of HPV lesions and cervical cancer: Gynecology and Obstetrics Unit, Rodolico University Hospital, Department of General Surgery and Medical-Surgical Specialties of the University of Catania; Gynecological Oncology Unit, Humanitas and Colposcopy Unit, Humanitas Medical Care, Catania; Colposcopy Unit, Humanitas Medical Care, Catania, Italy. We enrolled all the cases of women treated for CIN3 for which we knew the results of the HPV test, genotyping, histological examination of the cone, and the state of the margins, as well as the treatment and the follow-up to which they were subjected. Women who underwent LEEP for pathology other than CIN3, who did not have follow-up data, or who had a history of cervical carcinoma were excluded.

Of the initial 491 patients, 40 women were diagnosed with a diagnosis other than CIN3 and data on 37 women were incomplete, so only 414 women met the inclusion criteria.

The histological slides were diagnosed according to the WHO classification as CIN2+ for all the cases of CIN2, CIN3, and SCC lesions.

The following clinical data of the patients were collected: age, HPV genotype, histological examination of the cone, and type of therapy.

All slides were reviewed by two dedicated pathologists; cone length, the presence of concomitant AIS or AC glandular pathology, and the status of the resection margin were considered. Margin status was considered positive if any margin (ectocervical, endocervical, or deep/circumferential) was involved, and negative if all margins were histologically free of neoplasia.

**Figure 1:**
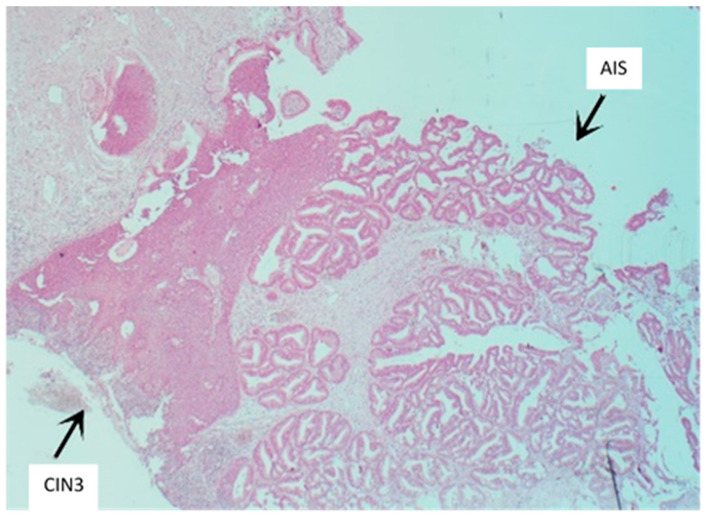
Squamocolumnar junction: combined presence of cervix adenocarcinoma in situ and CIN3 lesion, magnification 250×.

The study sample consisted of a group of women with a histological diagnosis on the LEEP cone of CIN3 lesion and a group of women with a histological diagnosis of a CIN3 lesion coexisting with AIS or AC (Figure 1).

**Figure 2:**
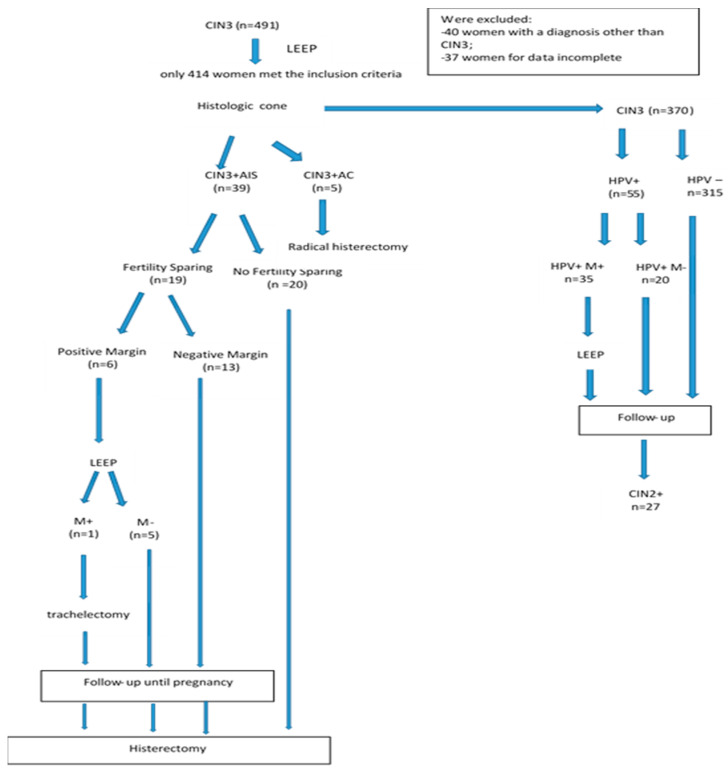
Flowchart of study population.

Figure 2 shows the flow diagram of the study population.

### 2.1. HPV Test and Genotyping

After cytological sampling for HPV DNA, samples were sent to the laboratory for DNA extraction and viral DNA genotyping via genetic amplification, followed by hybridization with genotype-specific probes capable of identifying most of the HPV genotypes of the genital region—13 high-risk HPV genotypes (16, 18, 31, 33, 35, 39, 45, 51, 52, 53, 56, 58, 59), 11 low-risk genotypes (6, 11, 40, 43, 44, 54, 70, 66, 68, 73, 82), and 3 undefined-risk genotypes (69, 71, 74). The commercial method used was the MAG NucliSenseasy system (bioMérieux SA, Marct l’Etoile, France). The technique used was described previously [17].

### 2.2. Colposcopy

Colposcopy was performed using a Zeiss OPM1F colposcope (Carl Zeiss, Jena, Germany). We evaluated the visibility of the squamocolumnar junction (SCJ) and studied the reactivity of the squamous epithelium to the application of acetic acid before and after adding Lugol’s solution. Afterward, we used the nomenclature proposed by the International Federation for Colposcopy and Cervical Pathologies (IFCPC) in three grades of abnormality increasing according to severity: (i) abnormal transformation zone (ATZ) grade 1 (ATZ1); (ii) grade 2 (ATZ2); or (iii) cancer. If lesions were evident, targeted biopsies were performed in sections.

### 2.3. LEEP Technique

The electrosurgical excision procedure with loop (LEEP) was performed with colposcopic guidance under local anesthesia in an outpatient clinic by an expert colposcopist.

The size of the loops depended on the characteristics of the lesion and the shape of the cervix.

Resection margins were maintained 2–3 mm beyond the lesion, and completeness of lesion removal was verified colposcopically. Fractional histologic examination of all removed tissue allows for definitive diagnosis and follow-up decisions.

Cone margins were reported as positive if the distance between the lesion and the resection surface was <1 mm.

Residual disease is the diagnosis of CIN2+ at the first follow-up after LEEP; low-grade cervical lesions were not considered as residual disease.

Recurrence or relapses occur when the CIN2+ lesion is diagnosed after a negative test.

The presence of the same HPV genotype before and after LEEP was considered to be HPV-persistent.

The first follow-up check is carried out six months after LEEP.

The follow-up protocol involved a co-test and colposcopy every 6 months for two years and annually thereafter; if three successive co-tests were negative, the frequency changed to every three years.

For patients who undergo fertility-sparing management, the following is prescribed: co-testing and endocervical curettage every 6 months for at least 3 years, and then annually or until hysterectomy is performed.

The follow-up of women with adenocarcinoma undergoing radical hysterectomy with bilateral lymphadenectomy consisted of clinical monitoring evaluated according to the National Comprehensive Cancer Network (NCCN) criteria.

Written informed consent was obtained from all participating patients regarding the use of the data for scientific purposes.

The University Hospital’s ethics committee waived the requirement for ethical approval and informed consent because this study used previously archived data, according to current legislation (20 March 2008), AIFA.

### 2.4. Statistical Analysis

The analyses were conducted with the STATA software version 13 (StataCorp version 13, College Station, TX, USA). Descriptive statistics were expressed as frequency, arithmetic mean, and standard deviation. The results are summarized in tables and figures. The relationship between categorical variables was assessed. We compared the frequency of specific HR HPV genotypes in patients with a coexisting CIN3 with AIS lesion or CIN 3 alone. A statistical analysis including independent χ2, and *t*-tests was performed, considering all *p* values < 0.05 to be significant.

## 3. Results

The study sample consisted of a group of 370 (89.4%) women with a single CIN3 and a group of 44 (10.6%) women with CIN3 coexisting with AIS or AC. Patients’ characteristics are shown in Table 1.

The mean height of the cone specimen was 15.5 mm (SD ± 5.7, range 5.7–25.0 mm). The surgical margins of cervical conizations were free of disease in 82% of cases.

The mean age of women with a single CIN3 was 35.6 years (28–59). At the first post-conization evaluation, we had viral clearance in 85.1% (315/370) of cases. Thirty-five (9.4%) women had positive margins and viral persistence. Subjected to a second LEEP, they achieved negative margins and viral clearance (Table 1).

Among the 44 women with CIN3 coexisting with glandular disease, a total of 39 (88.7%) cases of AIS and 5 (11.3%) cases of adenocarcinoma (usual type, mucinous) were identified (Table 1). The mean age of women diagnosed with AIS or AC was 39.1 years (SD ± 9.3, range 25–61 years), and only four women were post-menopausal.

All considered women were HPV-positive. In patients with a single CIN3, 11 high-risk genotypes were detected. In patients with CIN3 associated with AIS or AC, only four different genotypes were detected: HPV16, HPV18, HPV45, and HPV33 (Table 2).

The most frequent genotypes in women with a single CIN3 were HPV16 (39.7%), HPV31 (19.4%), and HPV18 (11.3%), followed by HPV33 (8.9%) and HPV45 (4%). The other genotypes were HPV 33, 35, 51, 52, 56, 58, and 66.

In CIN3 patients with coexisting AIS, we had 17 (38.6%) cases of the HPV16 infection, 18 cases (41%) with genotype 18, and 4 cases (9%) with HPV45. Multiple infections 16 and 33 were present in two women. Genotype 33 was found only in multiple lesions.

Overall, the frequency of HPV 18 was significantly higher in CIN3 lesions coexisting with AIS compared to solitary CIN3 lesions, χ2 = 27.73 (*p* < 0.001), while the frequency of other high-risk genotypes was significantly higher in patients with a single CIN3 compared to patients with CIN3 coexisting with AIS (Table 3).

Of the 44 women with coexisting glandular disease, 40 were of childbearing age, of whom 19 were desiring offspring, and 4 women were in menopause.

The five women with adenocarcinoma (AC), whose average age was 47 years, were treated with radical hysterectomy (Figure 2). In particular, we had a case of stage IA1 microinvasive carcinoma with positive LVSI and positive sentinel lymph node, one IA2 case, two cases at stage IB1, and one IB2 case. They are all cases of stage I early cancer. All were treated with type b radical hysterectomy (nerve sparing) according to the Querleu-Morrow classification with bilateral pelvic lymphadenectomy; however, in case IB2, the involvement of the parameters (re-staging IIB) made adjuvant chemotherapy necessary. The follow-up consisted of clinical monitoring that was evaluated according to the National Comprehensive Cancer Network (NCCN) criteria. It is now recognized that the histological cell type (squamous or glandular) has no impact on survival for stage I diseases; to date, after 4 years of follow-up, the women from this study are disease free.

**Figure 3:**
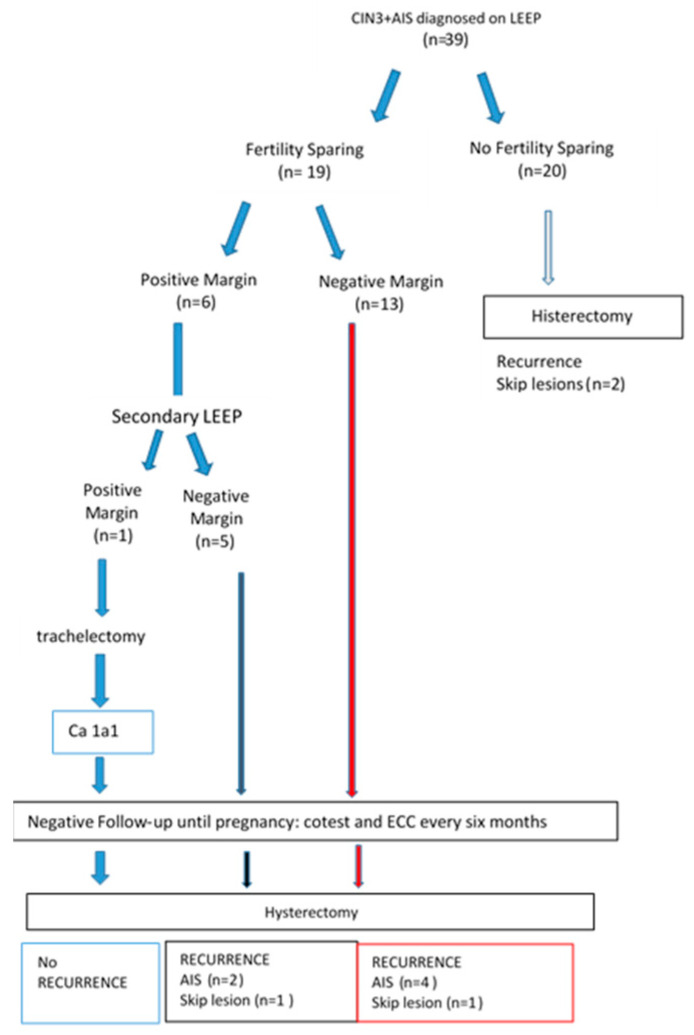
The therapeutic options, follow-ups, and the recurrence for the fertility-sparing cases and non-fertility preservation cases of the study population. The different color of the box indicates the groupings of the cases under study.

The women in the group comprising CIN3 coexisting with AIS, who had already completed pregnancy (*n* = 20), underwent subsequent hysterectomy, as recommended in the 2019 ASCCP guidelines. The histological result highlighted two cases of skip lesions (Figure 3).

In the fertility-sparing group (*n* = 19), six women with surgical involvement of the margins underwent further conization to obtain complete excision of the lesion. One of these women subsequently underwent simple vaginal trachelectomy due to the persistence of the positive margin. We have saved a percentage of healthy stroma for greater pregnancy success and to avoid the risk of cervical incompetence, premature birth, premature rupture of membranes, and other infectious conditions. The histological outcome showed a stage 1A1 adenocarcinoma of the endocervical type with a clear margin and without invasion of the lymphovascular space. Follow-up tests were performed quarterly in the first year and then every 6 months in the following year. After pregnancy, which began after 22 months, she underwent a total hysterectomy without bilateral adnexectomy (Figure 3).

All 19 women, eager to have children, with CIN3 coexisting with AIS and negative margins, underwent fertility-sparing procedures. After obtaining informed consent, they underwent a follow-up, the average duration of which was 27 months (range 6–52 months) with a co-test, colposcopy, and endocervical curettage. They underwent simple hysterectomy at the end of pregnancy, as recommended. Histological examination of the uterus revealed the presence of eight cases (18.2%) of recurrent disease, and the lesions were multifocal (skip lesions) in two cases (Figure 3).

In total, the 39 women with CIN3 coexisting with AIS all underwent hysterectomy; the histological examination highlighted 10 (22.7%) cases of relapses, of which 8 cases were present in women with preserved fertility, and 4 (9%) cases were skip lesions. Furthermore, CIN3 coexisting with AIS showed a higher persistence rate than squamous disease (Table 1).

## 4. Discussion

In our case series, a relatively high-rate percentage (10.6%) of mixed lesions (AIS and AC) emerged in women undergoing CIN3 conization, and all cases of coexisting glandular pathology detected were mucinous, with HPV being correlated. The literature reports a variable rate between 7.7% and 10% of glandular lesions in women with CIN3 [15,17]. More than half of AIS cases are detected with coexisting HSIL or invasive SCC [15].

Usually, the AIS does not exceed 15 mm in length; thus, preinvasive glandular lesions are, in most cases, “masked” by the more evident squamous lesions. It is known that the extension to the surface and in the AIS glandular crypts is proportional to a woman’s age, with it being 5.6 mm on average in women under the age of 36 and 10.8 mm over 36 years of age [18]. Also, the depth of the involvement of the crypt is linked to age, with a higher superficial involvement in young women [19]. When these pictures, which are difficult to recognize even when isolated, are present in a mixed lesion, it is easy to understand how it is possible to highlight only the squamous lesion, which is more visible and recognizable [15]. When there is a mixed pathology (squamous and glandular)—both cytologically and colposcopically—the squamous lesion is, in most cases, the only one to be highlighted, and the targeted biopsy confirms the presence of the squamous lesion alone. Only excisional therapy (which should therefore be encouraged over destructive therapy) highlights the association of an AIS. Therefore, when a glandular lesion is suspected due to a positive cytology, even when the targeted incisional biopsy does not detect a glandular lesion, excisional therapy should still “always” be performed with wide margins for the possibility that an AIS or AC is present in the immediate vicinity and for the evaluation of a possible stromal invasion (measurable) or involvement of the lymphovascular spaces to be carried out. 

In recent decades, several theories have been proposed concerning the origin of combined CIN3 and AIS lesions. The combined CIN3 and AIS lesions appear to arise from the same progenitor cells located at the squamocolumnar junction. More specifically, persistent infection with HPV16, 18, and 45 in association with potential cofactors can activate reserve cells that, having bipotential and being undifferentiated, can differentiate into a squamous or glandular cell. Glandular metaplasia is very rare and can give rise to atypical simple glandular metaplasia, tubal glandular metaplasia (ciliated cells), or intestinal glandular metaplasia (goblet cells), causing the onset of the various forms of HPV-related AIS—simple, tubal, or intestinal, respectively. Atypical squamous metaplasia may occur simultaneously with glandular metaplasia and may result in CIN3, giving rise to a “mixed disease” [15].

Moreover, recent studies suggest that the HPV genotype involved in mixed disease is the same for both coexisting diseases. Kawakami et al. [20] examined eight cases of H-SIL (CIN3) combined with AIS and examined HPV subtypes, HPV16 L1 gene methylation status, and the Krt7 immunohistochemical staining pattern. Of these cases, 75% showed the same HPV subtype in both HSIL and AIS, mainly HPV16 (62.5%). Another very recent study from 2023 [21] reports the coexistence of three different histological types: poorly differentiated adenosquamous carcinoma (glassy cell carcinoma), adenocarcinoma of the usual type, and a CIN3. PCR isolated only HPV type 18. Analysis of the HPV fragments isolated from the patient showed that all carcinomas had the same HR HPV genotype (HPV18).

In the literature, there is a clear difference between HPV genotypes affecting glandular disease and those infecting squamous epithelium; while HPV16 causes the majority of squamous cell carcinomas, genotypes 18 and 45 are found in a higher percentage of adenocarcinoma than squamous cell carcinomas [4,13,22,23,24].

Some researchers hypothesize that coexisting glandular and squamous lesions, in addition to sharing a common etiology, have a different biological behavior from the single squamous lesion (CIN3), and the study of the different genotypes that are identified in the context of the lesions seems to confirm this [25].

In our study, women with coexisting CIN3 and AIS had a significant prevalence of HPV18 (*p* < 0.001), while the association with all other genotypes, including HPV16, was not significant.

In addition, we also found a difference in the frequency of genotypes in the two groups. In patients with a single CIN3, we found the frequency of 11 genotypes; in women with CIN3 associated with AIS, we isolated only 4 different genotypes: HPV16, 18, 33, and 45. This is also confirmed by other authors. In particular, a study [25] investigated the frequency of these genotypes in patients with CIN3 and AIS, in women with a single CIN3, and in women with a single AIS. This study showed that there were no significant differences in the frequency of HPV genotypes 16, 18, and 45 between patients with coexisting CIN3 and AIS vs. patients with a single AIS, but there was a difference in the group comprising women with a single CIN3. These results suggest that coexisting CIN3 and AIS lesions have a biological behavior much more similar to that of a single AIS and one that is different from solitary CIN3 lesions. All this also justifies the different treatment to which coexisting CIN3 and AIS lesions are directed; in fact, the latter are treated with hysterectomy, while CIN3 is treated only with conization.

Biological features such as multifocality, localization in the endocervical canal, and their association with occult invasive adenocarcinoma mean that the standard treatment for cervical AIS is hysterectomy, which is a more aggressive treatment than the conization that is used for CIN3 [26,27].

In fact, due to skip lesions, residual glandular neoplasms may be present even in cases of apparently negative surgical margins in conservatively treated cases. In our study, 9% of cases of CIN3 associated with AIS were skip lesions. Skip lesions, which occur in 6.5–15% of AIS lesions, provide a reduced predictive value of negative margins because of the absence of residual disease. 

None of the women with a single CIN3 in our study required hysterectomy. The 35 patients (9.4%) with residual disease underwent a second LEEP. The recurrence rate was 7.3%.

The current trend of delaying pregnancy among women has led to an increase in fertility-sparing procedures, such as cold blade conization (CKC) or LEEP, to treat lesions.

The 2019 ASCCP guidelines define optimal cervical cone excision as a single, oriented cone at least 10 mm long in a woman with a desire to bear children or 20 mm in a woman with children. They consider top-hat conization—i.e., the procedure that involves two stages of excision of the loop: a conventional LEEP followed by a second excision of the endocervix with a smaller loop—to be unacceptable [26,27].

Although the Society of Gynecologic Oncology (SGO) and ASCCP management guidelines allow conservative management of patients with AIS who desire to become pregnant with negative conization margins, patients should be advised that the risk of residual AIS (20%) and even invasive adenocarcinoma (3%) is not negligible.

In our study, the histological examination of the uterus removed in women with CIN3 with associated AIS highlighted 10 cases of recurrent disease (22.7%), of which 8 occurred in women with fertility sparing; in 4 cases (9%), recurrent disease was represented only by AIS in its multifocal form (skip lesions). Furthermore, our study also highlights a high degree of persistence of the HPV infection in high-grade glandular pathology compared to squamous disease (Table 1). Studies report that the clearance of HPV in AIS takes longer than in squamous lesions; therefore, a longer follow-up is necessary [26,27].

For patients with negative margins undergoing fertility sparing, a follow-up must be foreseen with a co-test and a study of the cervical canal using brush cytology and curettage must take place every six months for the first 3 years, and then every year for the next two years or until hysterectomy.

Given the high rate of HPV18-positive AIS cases, endocervical sampling for any patient who tests positive for HPV18 is suggested.

In our opinion, however, based on what has been explained so far, the evaluation of the cervical canal should be carried out in all cases of CIN confirmed by targeted biopsy and, mainly, in all those to be subjected to excisional therapy to search for possible lesions associated with the cervical canal and to decide the depth of the sample to be removed [28]. It should be emphasized that none of the available investigations for the evaluation of the endocervical canal have a good diagnostic accuracy for glandular lesions. The conization of the cervix and histopathological confirmation are essential for the definitive diagnosis of mixed lesions (CIN3 and coexisting AIS) whose glandular component provides a worse prognosis for the lesion than for a single CIN3. Our study confirms the multifocal biological nature of the CIN3 lesion coexisting with AIS compared to the single CIN3 lesion. The isolation of only four HPV genotypes, the prevalence of genotype 18, the presence of skip lesions (9%), and occult invasive adenocarcinoma (one case of 1A1 adenocarcinoma) in the deep fissures of the endocervical glands, as well as a higher rate of viral persistence and recurrence make the mixed lesions more similar to a glandular pathology than to a squamous one. These characteristics make hysterectomy necessary and cervical conization alone insufficient, which is instead considered the optimal treatment method for a single CIN3. 

Women with a history of cervical dysplasia have an increased risk of developing vaginal dysplasia, and surgical management must consist of follow-ups for at least 25 years. According to ASCCP risk-based management consensus guidelines, women that check these criteria have to be subjected to a follow-up with vaginal colposcopy and co-test [29,30].

Current vaccines targeting HPV 16 and 18 with cross-protection against HPV 45 are expected to have good protection against HPV-related glandular disease [31].

The limitations of our study include its retrospective nature, which does not allow a complete analysis to be carried out due to the lack of data, such as the deficit in cytological samples or biopsies performed before LEEP. Another limitation is represented by the small number of cases studied, which is inevitably linked to the rarity of the pathology in question. The strengths of our study consist of its multicenter design and of the homogeneity of the included patients. Furthermore, the inclusion of a second histopathological review to confirm the diagnosis in this article further strengthens the validity of the results. The therapeutic options of the entire study, the follow-ups, and the benefits of vaccination for the population were illustrated.

## 5. Conclusions

This study of specific HPV genotypes highlighted a significantly different frequency of genotypes for patients with CIN3 associated with AIS and for patients with a single CIN3, leading to the suspicion of there being a different biological behavior between the mixed lesion and a single CIN3. Our study confirms the different biological nature of CIN3 coexisting with AIS compared to the single CIN3 lesion. The clinical differences highlighted in our study make the mixed lesions more similar to a glandular pathology than to a squamous one. All this makes hysterectomy necessary as a treatment and makes cervical conization insufficient, which is considered the optimal treatment method for a single CIN3.

## Figures and Tables

**Table 1:** Patients’ characteristics.

	CIN3 *n* = 370	CIN3+ AIS-AC *n* = 44
Independent Variables	*n*	*n*
Age	35.6 years	39.1 years
Menopause	55/370 (14.8%)	4/44 (9%)
Desiring offspring	140/370 (37.8%)	19/44 (43.2%)
Completed childbearing	175/370 (47.3%)	21/44 (47.7%)
HPV genotypes	11	4
HPV 16	147/370 (39.7%)	17/44 (38.6%)
HPV18	43/370 (11.6%)	18/44 (40.1%)
Positive margins	67/370 (18.1%)	6/44 (13.6%)
HPV persistence	55/370 (14.9%)	41/44 (93.2%)
Skip lesions	0	4/44 (9%)
Second LEEP	35/370 (9.4%)	6/6 (100%)
Trachelectomy	0	1/6
Hysterectomy	0	39/39 (100%) CIN3+ AIS
Radical Hysterectomy	0	5/5 (100%) AC
Recurrence	27 (7.3%)	10 (22.7%)

**Table 2:** Frequency of genotypes in patients with CIN 3 compared to patients with CIN3 coexisting with AIS.

	CIN3		CIN3-AIS/AC	
Genotype HPV	*n*	%	*n*	%
**16**	147	39.7	17	38.6
**31**	72	19.4	0	0
**18**	42	11.3	18	41
**33**	33	8.9	2	4.5
**45**	15	4	4	9
**51**	15	3.8	0	0
**52**	16	4.3	0	0
**35**	11	3	0	0
**56**	10	2.7	0	0
**58**	5	1.4	0	0
**66**	4	1	0	0
	370	(89.4%)	44	(10.6%)

AIS: adenocarcinoma in situ; AC: adenocarcinoma; CIN3: cervical intraepithelial neoplasia.

**Table 3:** The frequency of HPV 18 is significantly higher in CIN lesions coexisting with AIS than in a single CIN3.

	CIN3		CIN3-AIS/AC					
HPV Genotype	*n*	%	*n*	%	OR	CI 95%	*p*	χ2
16	147	39.7	17	38.6	0.96	0.50–1.81	0.89	0.020
18	42	11.3	18	41	5.41	2.74–10.69	0.0001	27.73
33	33	8.9	2	4.5	0.66	0.15–2.87	0.57	0.31
45	15	4	4	9	2.37	0.75–7.48	0.13	2.28

AIS: adenocarcinoma in situ; AC: adenocarcinoma; CIN3: cervical intraepithelial neoplasia.

## Data Availability

Data are contained within the article.
